# Determining Prenatal, Early Childhood and Cumulative Long-Term Lead Exposure Using Micro-Spatial Deciduous Dentine Levels

**DOI:** 10.1371/journal.pone.0097805

**Published:** 2014-05-19

**Authors:** Manish Arora, Christine Austin, Babak Sarrafpour, Mauricio Hernández-Ávila, Howard Hu, Robert O. Wright, Martha Maria Tellez-Rojo

**Affiliations:** 1 Department of Preventive Medicine, Icahn School of Medicine at Mount Sinai, New York, New York, United States of America; 2 Faculty of Dentistry, University of Sydney, Sydney, Australia; 3 Office of the Director, National Institute of Public Health, Cuernavaca, Morelos, México; 4 Dalla Lana School of Public Health, University of Toronto, Toronto, Ontario, Canada; 5 Division of Statistics, Center for Evaluation Research and Surveys, National Institute of Public Health, Cuernavaca, Morelos, México; Stony Brook University, Graduate Program in Public Health, United States of America

## Abstract

The aim of this study was to assess the validity of micro-spatial dentine lead (Pb) levels as a biomarker for accurately estimating exposure timing over the prenatal and early childhood periods and long-term cumulative exposure to Pb. In a prospective pregnancy cohort sub-sample of 85 subjects, we compared dentine Pb levels measured using laser ablation-inductively coupled plasma mass spectrometry with Pb concentrations in maternal blood collected in the second and third trimesters, maternal bone, umbilical cord blood, and childhood serial blood samples collected from the ages of 3 months to ≥6 years. We found that Pb levels (as ^208^Pb:^43^Ca) in dentine formed at birth were significantly associated with cord blood Pb (Spearman ρ = 0.69; n = 27; p<0.0001). The association of prenatal dentine Pb with maternal patella Pb (Spearman ρ = 0.48; n = 59; p<0.0001) was stronger than that observed for tibia Pb levels (Spearman ρ = 0.35; n = 41; p<0.03). When assessing postnatal exposure, we found that Pb levels in dentine formed at 3 months were significantly associated with Pb concentrations in children’s blood collected concurrently (Spearman ρ = 0.64; n = 55; p<0.0001). We also found that mean Pb concentrations in secondary dentine (that is formed from root completion to tooth shedding) correlated positively with cumulative blood lead index (Spearman ρ = 0.38; n = 75; p<0.0007). Overall, our results support that micro-spatial measurements of Pb in dentine can be reliably used to reconstruct Pb exposure timing over the prenatal and early childhood periods, and secondary dentine holds the potential to estimate long-term exposure up to the time the tooth is shed.

## Introduction

**Figure 1:**
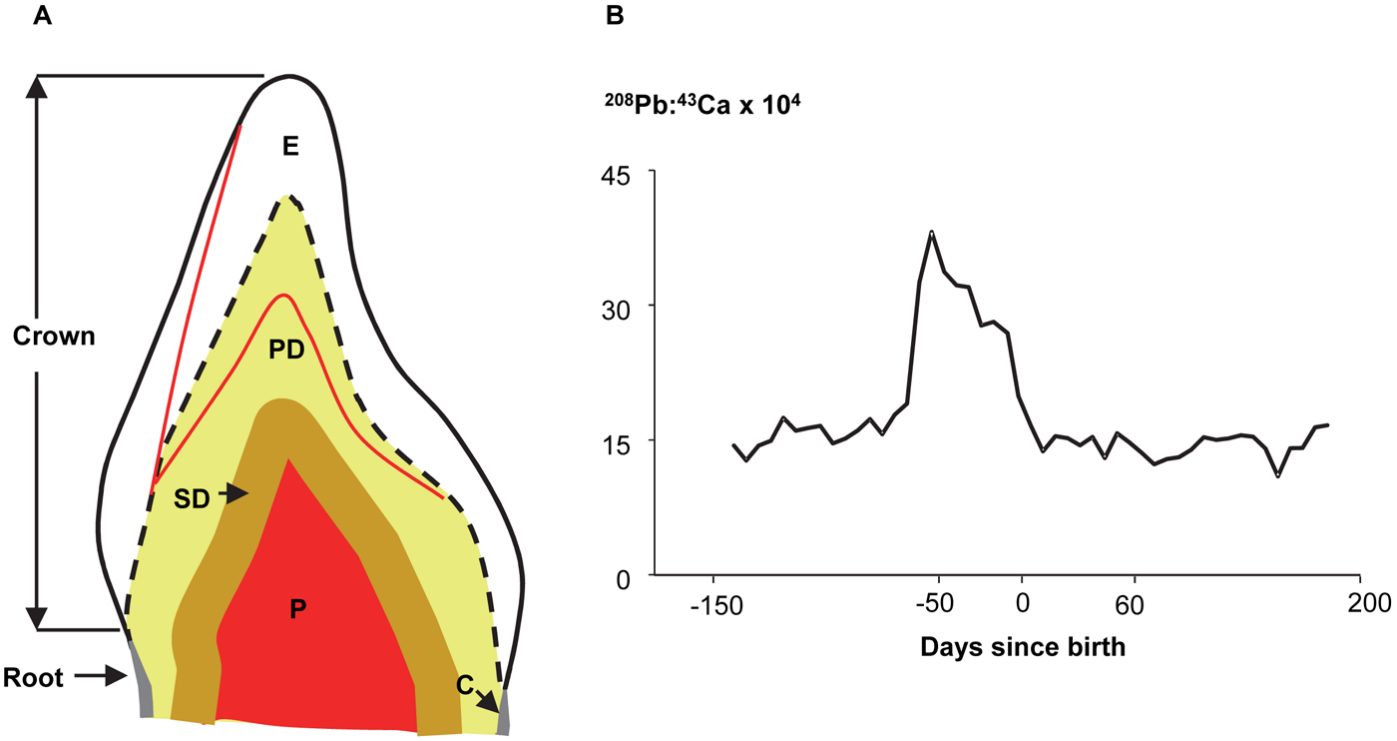
Overview of the dentine Pb biomarker. (**a**) Schematic of a deciduous human incisor. During tooth formation the deposition of the enamel (E) and primary dentine (PD) matrix commences at the enamel-dentine junction (dashed line). At birth the neonatal line (red line) is formed in enamel and dentine providing a landmark to distinguish prenatally formed parts of teeth from those formed postnatally. In our analysis, prenatally formed primary dentine adjacent to the enamel-dentine junction is sampled to obtain prenatal Pb exposure information. Sampling points in dentine at the neonatal line measure fetal Pb exposure at the time of birth. Secondary dentine (SD) formation starts after the completion of the tooth root and proceeds at a slower rate as long at the tooth remains vital. Measurements in this region are used to estimate cumulative long-term Pb exposure from root completion to the time tooth was shed. Pulp chamber (P) and cervical margin (C) between the tooth crown and root are also indicated. (**b**) Lead distribution and developmental timing of primary dentine sampled in a deciduous molar. It is our hypothesis that Pb levels at each dentine sampling point represent Pb exposure experienced when that part of dentine was being mineralized. In this individual dentine Pb levels showed a marked rise prior to birth. Key time points for change in dentine Pb levels are shown; other points on the graph can be similarly dated.

Susceptibility to environmental toxicants is heightened during prenatal and early childhood periods when many systems are developing and vulnerable to the disruptive effects of chemicals [1]. However, accurate objective assessment of exposure timing, especially during fetal development, remains a major challenge in environmental epidemiologic research. This primarily arises from the absence of direct fetal biomarkers of exposure that can be safely used in large study populations. In earlier work, we used naturally shed deciduous tooth dentine to uncover early life exposure to metals including Pb, Mn, Ba and Sr [2]–[6]. We combined micro-spatial elemental analysis of teeth with detailed histological techniques to construct a detailed temporal ‘map’ of exposure during the prenatal and early childhood periods. Figure 1 depicts the basic structure of a tooth crown and provides an example of our approach to estimating exposure timing on a fine scale over the prenatal and early postnatal periods. A brief description of dental anatomical terminology used in this manuscript is given in Table S1 (see Information S1).

Although the use of teeth to obtain cumulative metal exposure information has been proposed for many decades [7]–[10], the validity of obtaining exposure *timing* from dentine remains to be adequately tested. There exists a need to validate the temporal Pb exposure information obtained from dentine against Pb concentrations in other established biomarkers including maternal blood and bone, cord blood and serial childhood blood Pb levels. Furthermore, comparing the cumulative Pb exposure information from secondary dentine with another measure of integrated long-term Pb exposure such as the cumulative blood Pb index (CBLI) [11],[12] would provide evidence that life-long exposure can also be estimated from dentine.

In the study presented here, we evaluate if the dentine biomarker accurately measures (i) the intensity and timing of fetal exposure, (ii) the intensity and timing of early childhood exposure, and (iii) cumulative long-term exposure. We undertook this study in a prospective mother-child cohort where we measured Pb in umbilical cord blood collected at birth, and in serial venous blood samples collected at approximately 6- to 12-monthly intervals from the age of 3 months to 6 years, and then measured blood Pb again between the ages of 7 to 11 years. We also measured maternal blood Pb in each trimester of pregnancy and at multiple time points after the birth of their child. Importantly, we have measured Pb in the bones of the mothers which allowed us to explore the association of maternal Pb body burden with our tooth Pb biomarker.

## Methods

### Study Population

Mother–child pairs in this study were drawn from the longitudinal birth cohort studies in Mexico City that comprise the Early Life Exposures in MExico and NeuroToxicology (ELEMENT) project. Subjects were originally recruited between 1994 and 2003 to investigate the long-term consequences of prenatal environmental factors on child development [13]–[15]. Detailed information on the study design and data collection procedures has been published previously [13], [15], [16]. Mothers were recruited during pregnancy and maternal venous blood was sampled once each during the first, second and third trimesters. Anthropometric data from the mother and newborn, and umbilical cord and maternal venous blood samples were gathered within 12 hr of delivery. Information on estimated gestational age, based on the date of last menstrual period, and characteristics of the birth and newborn period were extracted from the medical records. Exclusion criteria included factors that could interfere with maternal calcium metabolism; medical conditions that could cause low birth weight; prematurity (<37 weeks) or an infant with Apgar score at 5 min of≤6, a condition requiring treatment in neonatal intensive care unit; or serious birth defects; psychiatric illness, seizures, or kidney or cardiac disease; preeclampsia, systolic BP>140 mmHg or diastolic BP>90 mmHg; gestational diabetes; consumption of alcoholic beverages; addiction to illegal drugs; and continuous use of corticosteroids. The mother–child pairs were contacted and recalled for the follow-up assessment between 2008 and 2010 when the children were 7–15 years of age, and tooth collection was incorporated into this visit. Only one child for each mother was included in this study, regardless of birth order. Medical history, physical examination, and venous blood samples were collected from or performed on mothers and children.

### Ethics Statement

All participating mothers signed a written consent form, received a detailed explanation of the study intent and research procedures, as well as counseling on how to reduce environmental lead exposure. In addition, the children signed a written assent of minor form, easy to understand for them, and also received a detailed explanation of the study. All participants were encouraged to ask questions about the study in order to ensure their understanding. The research protocol was approved by the human subjects committees of the National Institute of Public Health of Mexico, the Harvard School of Public Health, Michigan School of Public Health, National Institute of Perinatology, and the American British Cowdray Medical Center (ABC Hospital).

### Collection and Analysis of Child and Maternal Samples

The sampling times of various biological media used in this study are shown in Table 1, and the distribution of Pb levels in these media is detailed in Table 2. Both median (range) and mean (SD) are provided to allow comparison with other studies in this cohort. Maternal tibia (cortical) and patella (trabecular) bone lead levels were measured within 1 month of delivery using a spot-source ^109^Cd K-shell X-ray fluorescence (K-XRF) instrument (ABIOMED, Danvers, MA, USA) [17]. Umbilical cord blood lead was analyzed using an atomic absorption spectrometry instrument (model 3000; PerkinElmer, Chelmsford, MA, USA). Child blood Pb was analyzed with the finger prick method using the LeadCare System (Magellan Biosciences, Chelmsford, MA, USA) at 3, 6, 18 and 30 months. At other ages venous blood Pb was measured using inductively coupled plasma mass spectrometry (Elan 6100; PerkinElmer, Norwalk, CT, USA). External blinded quality control samples (concentrations ranging from 2 to 88 µg/dL) were analyzed and provided by the Maternal and Child Health Bureau and the Wisconsin State Laboratory of Hygiene Cooperative Blood Lead Proficiency Testing Program. These analyses demonstrated good precision and accuracy with quality control specimens (Pearson r>0.98; mean difference <1 µg/dL). Additional details have been published previously [13], [15], [16].

**Table 1:** Timing of collection of Pb biomarkers used in the present study^a^.

Prenatal trimester				Birth	Postnatal months										
	1^st^	2^nd^	3^rd^		1	3	6	12	18	24	30	36	48	60	84+
Metal Biomarkers															
Fetal/cord blood				X											
Infant/child blood						X	X	X	X	X	X	X	X	X	X
Maternal blood	X	X	X												
K-XRF bone Pb					X										
Teeth															X

a additional measures are available in ELEMENT but not relevant for analyses presented in this study.

**Table 2:** Lead levels in biological media.

Matrix	n	Median Pb (range)
Prenatal dentine^a^	82	0.31 (0.06 to 3.99)
Postnatal dentine^a^	83	0.27 (0.06 to 2.72)
Secondary dentine^b^	84	0.01 (0.00 to 0.12)
Cord blood^c^	28	3.95 (0.90 to 14.7)^d^
Child blood at 3 mo^c^	55	3.50 (0.20 to 11.8)
Maternal blood during pregnancy^c^		
1^st^ trimester	51	5.70 (0.9 to 26.3)
2^nd^ trimester	52	4.70 (0.8 to 17.1)
3^rd^ trimester	49	5.20 (0.90 to 16.0)
Maternal bone^e^		
Tibia	43	6.45 (0.09 to 33.4)^f^
Patella	61	11.32 (0.01 to 42.9)^g^

a area under curve (AUC) of Pb levels in all sampling points within a region of dentine formed over a selected developmental period. Because the number of sampling points varies, AUC is adjusted for number of sampling points within that region.

b average of 7 measurements (as^ 208^Pb:^43^Ca).

c µg/dL.

d mean (SD) = 4.70 (3.72).

e µg Pb/g bone mineral.

f adjusted for negative values, mean (SD) = 8.95 (8.03).

g adjusted for negative values, mean (SD) = 12.91 (11.21).

Mothers were asked to provide their child’s naturally shed deciduous teeth. At the time of this study, we had 34 incisors, 25 canines and 26 molars that could be analyzed for metal concentrations. The teeth were stored dry at room temperature in packets provided by the research team, and were collected in person by a member of the field team. Information on the reason the tooth was shed, age at shedding, and if tooth was previously stored in any liquid was also collected from the mothers. Teeth were examined by a dentist for any visible defects including caries, attrition, cracks and discoloration. In the present study we use data from the 85 participants who donated deciduous teeth.

### Measurement of Pb in Teeth by LA-ICP-MS

Our approach to measuring metals in teeth using laser ablation-inductively coupled plasma mass spectrometry (LA-ICP-MS) and assigning developmental times has been detailed elsewhere (see Arora et al.[2], Hare et al.[6] and Austin et al.[5]). Briefly, we used the neonatal line (a histological feature formed in enamel and dentine at birth) and daily growth incremental markings to assign temporal information to sampling points.

The laser ablation unit used was a New Wave Research NWR-193 system (Kennelec Technologies, Mitcham, Victoria, Australia) equipped with a Nd:YAG laser emitting a nanosecond laser pulse in the fifth harmonic with a wavelength of 193 nm. The standard ablation cell was replaced with a Large Format Cell (LFC). The LFC has a large volume chamber capable of holding samples up to 15.2 cm^2^ in area. The *x-y-z* stage of the LFC employs a small volume ‘roving’ sampling cup that traverses the sample while the laser beam remains stationary. An approximately 40 cm length of Tygon® tubing (i.d. 3 mm) connected the laser ablation unit to an Agilent Technologies 7700cx (Agilent Technologies Australia, Forrest Hill, Victoria, Australia) ICP-MS. The instrument was fitted with a ‘cs’ lens system for enhanced sensitivity. The system was tuned daily for sensitivity using NIST SRM 612 (trace elements in glass). Polyatomic oxide interference was evaluated and minimized by monitoring the Th^+^/ThO^+^ (m/z 232/248) ratio. Typical oxide formation was consistently under 0.3%.

Using the laser we sampled 50 sampling points of 35 µm diameter in enamel and primary dentine adjacent to the enamel-dentine junction, and seven sampling points of 100 µm diameter in secondary dentine. Data were analyzed as ^208^Pb:^43^Ca ratios to control for any variations in mineral content within a tooth and between samples.

### Statistical Analysis

To test our hypothesis that dentine captures the timing and intensity of *prenatal Pb exposure* we undertook four comparisons. First, we studied the association of Pb levels in dentine sampling points at the neonatal line (a measure of fetal Pb exposure at birth according to our hypothesis) with Pb concentrations in umbilical cord blood, another marker of fetal Pb exposure. Second, we compared Pb levels in dentine formed at birth with blood Pb levels measured at different ages in childhood. If dentine indeed captures the timing of Pb exposure as we propose, the association of birth dentine Pb should be strongest with cord blood Pb and progressively weaker with Pb in blood samples collected at older ages. Third, we compared Pb levels in dentine formed during the second and third trimesters with maternal blood Pb levels collected at corresponding times during pregnancy. We hypothesized that prenatal dentine Pb should also be positively associated with maternal blood Pb during pregnancy because Pb levels in maternal and fetal circulation are strongly associated [18]. Fourth, we considered maternal bone Pb measurements at 1 mo postpartum which represent cumulative Pb exposure to the mother and a source of fetal exposure [14], [17], [19]. Because bone Pb represents integrated long-term exposure, we compared this maternal measure to the area under the curve (AUC) of Pb levels in all sampling points in prenatally formed dentine. K X-ray fluorescence provides a continuous, unbiased point estimate of the true bone lead measurement. However negative values are sometimes produced when the true values are below the detection limit of the instrument (in this study, 8 patella and 11 tibia Pb estimations, respectively, were below this limit). To avoid bias due to these negative values, we analyzed mean tibia and patella lead levels using both standard summary statistics that include the negative values and an estimation using interval regression, which simulates a normal distribution between 0 and the detection limit (2 µg Pb/g bone mineral), an approach we have used previously in this cohort [14]. To assess the potential influence of these negative values, we performed statistical analyses following two approaches: 1) including the negative estimates and 2) replacing them with new simulated values randomly generated with a uniform distribution between 0 and the lower limit of 2 µg Pb/g bone mineral. Since the results obtained were very similar (∼5 percent difference in the estimated coefficients), we present the results only from the former approach.

We also tested whether dentine captures *early childhood Pb exposure*. We compared Pb levels in dentine formed at 3 months postnatally with Pb concentrations in blood collected at this age. In our sample of incisors (teeth for which the coronal primary dentine completes at the earliest age) the most cervical dentine was formed between the ages of 1.2 to 3 months based on our histological analysis. Consequently, in some cases, the timing of Pb exposure represented by the dentine sampling points is not exactly concurrent with the 3-month blood Pb concentrations. However, it is important to consider that this disparity between the incisor dentine and blood measurements is likely to reduce the observed association between the two biomarkers and would not result in an inflated correlation (i.e. a bias towards the null). This issue was not of concern for canines and molars, where primary coronal dentine continues to form for 6 months or longer, and we were readily able to compare Pb levels in dentine formed at 3 months postnatally with 3-month blood Pb concentrations. We also extended this analysis by comparing Pb levels in these dentine sampling points with blood Pb levels measured at older ages in childhood.

Finally, we wanted to test the utility of secondary coronal dentine as a marker of *cumulative long-term Pb exposure*. This compartment of coronal dentine is formed from approximately 1.5 to 2.0 yrs for incisors and 2.5 to 3.0 yrs for canines and molars to the time of tooth shedding [20]. To test this hypothesis we compared the average Pb levels of seven sampling points in secondary dentine with CBLI. The CBLI is calculated by integrating multiple blood Pb measurements at different ages, and gives a single composite score that describes long-term Pb exposure (details on calculating CBLI and its interpretation have been presented by others [11], [12]). We excluded any participants who had less than 3 valid measurements of blood Pb from the ages of 1.5 years to the last included measurement which was 5 to 6 years for incisors and 7 to 11 years for canines and molars.

Spearman’s correlation analysis was used to measure the association of ^208^Pb:^43^Ca in teeth with blood and bone Pb measurements. When displaying bivariate associations as scatter plots with regression lines, we log_e_ transformed our data to achieve normally distributed variables. In some of our data analyses, we multiplied our dentine ^208^Pb:^43^Ca measurements with 10 or 10^4^ to avoid negative values during log_e_ transformation (and have indicated in the results when this was done). Results were considered statistically significant at *p*<0.05. All analyses were undertaken in STATA 10.0 (StataCorp, College Station, Texas).

## Results

**Figure 2:**
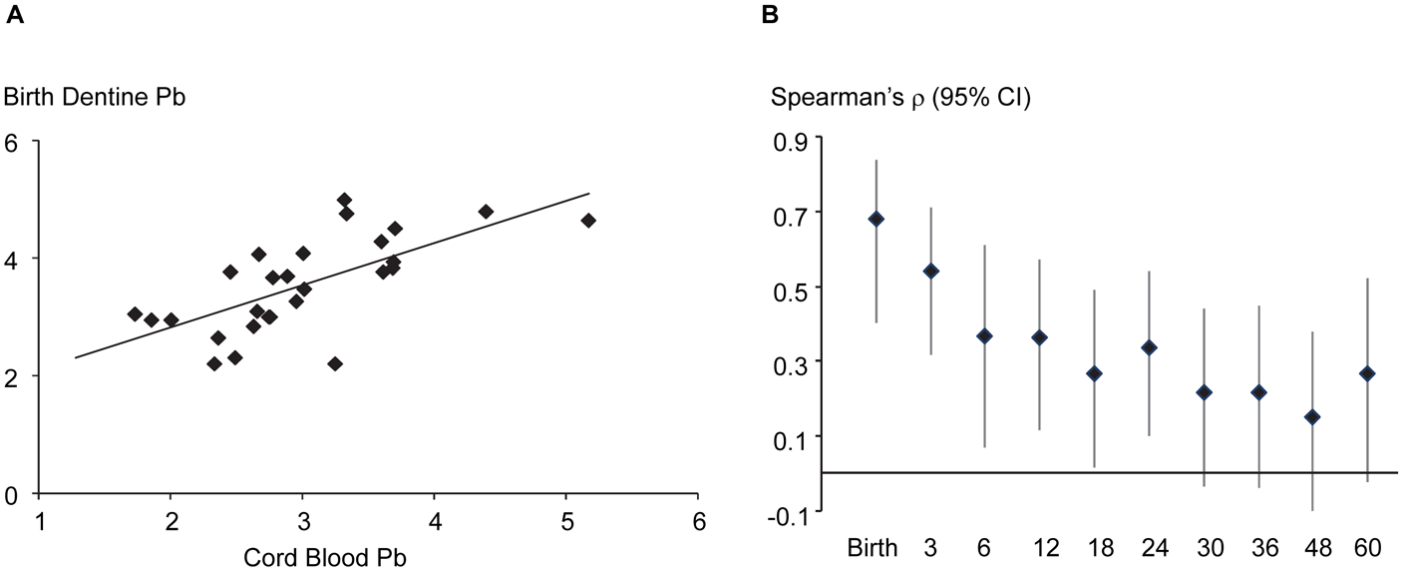
Association of Pb levels in dentine formed at birth with blood Pb concentrations at birth and childhood. (**a**) Significant positive association of Pb levels (as log_e_ [^208^Pb:^43^Ca10^4^]) in dentine at the neonatal line (representing fetal Pb exposure at birth) with umbilical cord blood Pb concentrations (as log_e_ [Pb×10 µg/dL]). (**b**) Spearman’s ρ (Y-axis; vertical lines show 95% CI) of Pb levels in dentine formed at birth with Pb concentrations in blood collected at different ages (X-axis; in months). The association of birth dentine Pb is strongest with umbilical cord blood Pb and is progressively weaker with blood sampled at later ages. Sample size at different time points: birth = 27, 3 m = 55; 6 m = 43; 12 m = 61; 18 m = 63; 24 m = 68; 30 m = 64; 36 m = 63; 48 m = 67; 60 m = 48.

When assessing the intensity and timing of prenatal exposure from dentine Pb, we found that Pb levels (as ^208^Pb:^43^Ca) in sampling points at the neonatal line (which represented approximately 12–15 days of dentine deposition around birth) were significantly associated with cord blood Pb levels (Spearman ρ = 0.69; n = 27; p<0.0001; Fig. 2a). Additional comparisons of Pb levels in these sampling points in dentine formed at birth with blood Pb concentrations measured later in life showed that the association continued to decline with increasing age at blood sampling (Fig. 2b.). Birth dentine Pb levels were not significantly associated with the child’s blood Pb sampled after 2 years of age.

**Figure 3:**
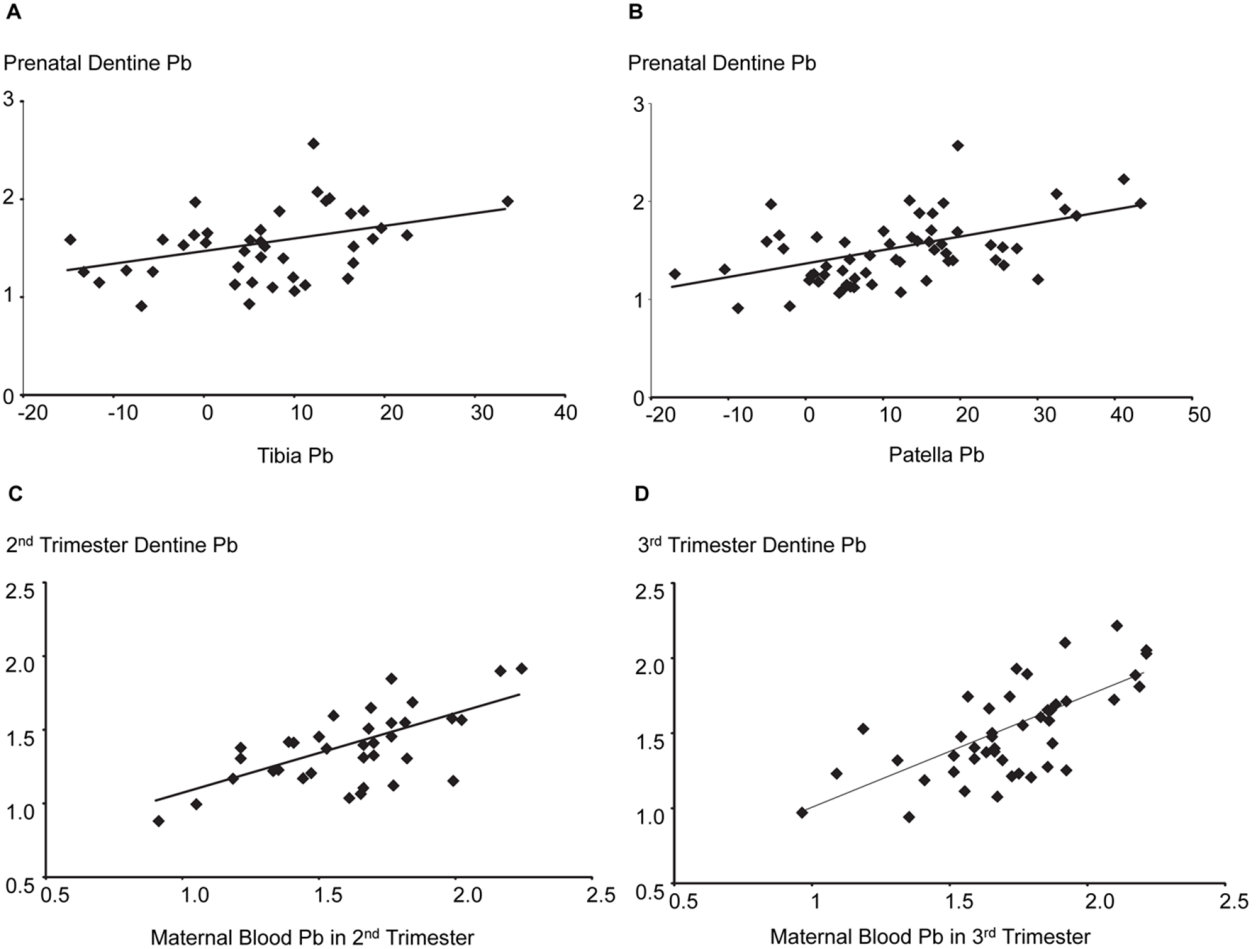
Association of Pb levels in prenatal dentine with maternal bone and blood Pb concentrations. Prenatal dentine Pb (as log_e_ [AUC ^208^Pb:^43^Ca×10^4^]) was positively associated with Pb concentrations (as µg Pb/g bone mineral) in **a**) tibia (Spearman ρ = 0.35; n = 41; p<0.03) and **b**) patella (Spearman ρ = 0.48; n = 59; p<0.0001). Relevance of negative bone Pb concentrations is discussed in detail by Hu et al.[17] Significant positive associations were observed between Pb concentrations in maternal blood and prenatal dentine formed during the **c**) 2^nd^ trimester (Spearman ρ = 0.60; n = 36; p<0.0001) and **d**) 3^rd^ trimester (Spearman ρ = 0.64; n = 44; p<0.0001).

Because Pb deposits in maternal bone are mobilized during pregnancy and serve as a major source of Pb exposure to the fetus [18], we compared Pb levels in prenatal dentine with maternal bone Pb measured at 1 month postpartum. Prenatal dentine Pb was significantly associated with both maternal tibia and patella Pb concentrations (Fig. 3a, b). However, the association with maternal patella Pb (Spearman ρ = 0.48; n = 59; p<0.0001) was stronger than that observed for tibia Pb levels (Spearman ρ = 0.35; n = 41; p<0.03). When we compared the Pb levels in dentine formed during the second and third trimesters with Pb concentrations in maternal blood collected concurrently, we also found significant positive associations (Second trimester: Spearman ρ = 0.60; n = 36; p<0.0001; Fig. 3c. Third trimester: Spearman ρ = 0.64; n = 44; p<0.0001; Fig. 3d).

**Figure 4:**
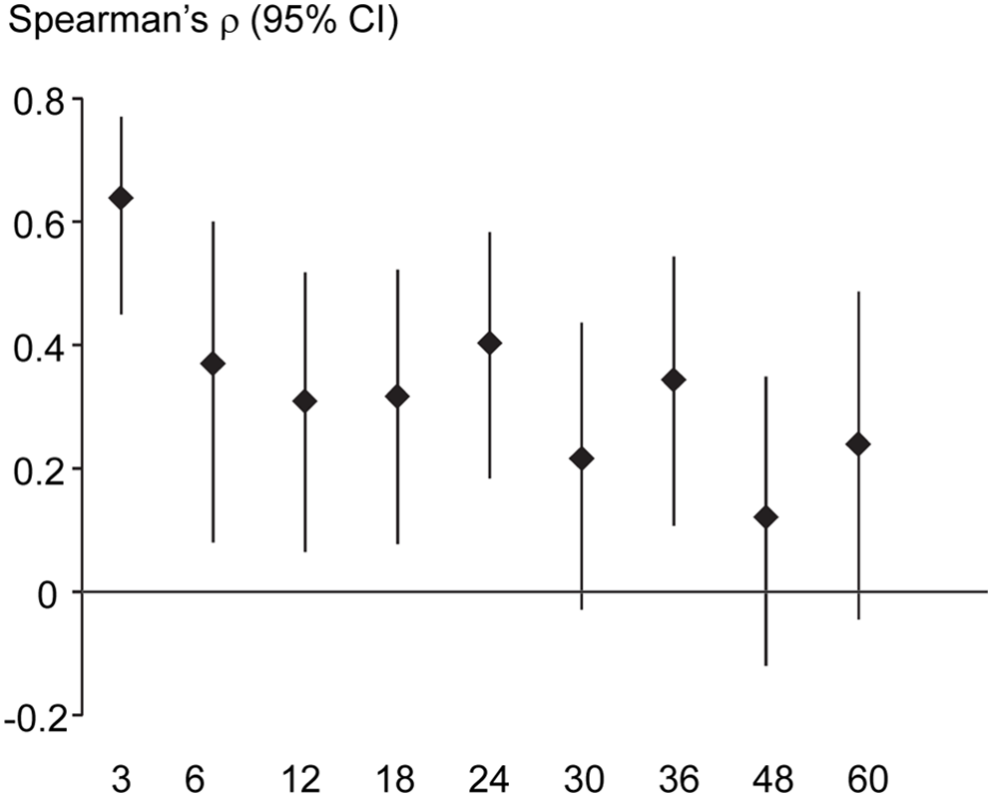
Timing of postnatal Pb exposure estimated from dentine. Spearman’s correlation coefficients (and 95% CI) for Pb levels in dentine formed at 3 months postnatally and child blood Pb also measured at 3 months. When compared to Pb levels in blood collected at older ages in childhood, the association was progressively weaker supporting the hypothesis that dentine retains the timing of postnatal Pb exposure.

To assess exposure timing in early childhood we compared Pb levels in dentine formed at approximately 3 months postnatally with Pb concentrations in the child’s blood sampled at 3 months of age. We found a significant positive association (Spearman ρ = 0.64; n = 55; p<0.0001). Furthermore, when we compared the association of Pb levels in these dentine sampling points (which were formed at approximately 3 months of age) with blood Pb concentrations measured at later ages we observed a progressively weaker association (Fig. 4).

To test the hypothesis that secondary coronal dentine captures long-term Pb exposure we compared the average of 7 sampling points in secondary coronal dentine of each tooth with CBLI which represents the integrated exposure in childhood (see Methods for additional details). We found that mean Pb concentrations (as ^208^Pb:^43^Ca) in secondary dentine correlated positively with CBLI (Spearman ρ = 0.38; n = 75; p<0.0007). Furthermore, when secondary dentine Pb levels were compared to Pb concentrations in maternal bone and maternal blood during pregnancy the correlations were weaker and statistically insignificant, supporting our hypothesis that secondary dentine Pb is a measure of cumulative postnatal exposure Pb exposure and not affected by prenatal Pb exposure (Table 3).

**Table 3:** Association of mean secondary dentine Pb levels (as ^208^Pb:^43^Ca) with cumulative blood lead index (CBLI), and Pb concentrations in maternal blood and bone.

Variable	Spearman’s ρ^a^
	n
	*p value*
CBLI^b^	0.38
	75
	*0.0007*
Second trimester blood Pb^c^	0.16
	52
	*0.25*
Third trimester blood Pb^c^	0.16
	49
	*0.26*
Patella Pb^c^	0.15
	61
	*0.24*
Tibia Pb^c^	0.06
	43
	*0.69*

a for correlation of secondary dentine Pb with variables listed.

b cumulative blood lead index (see Methods for additional details).

c maternal biomarkers.

## Discussion

Measuring exposure timing, especially during fetal development, is a major challenge in environmental epidemiology. Even when maternal biomarkers are available during pregnancy, they are not always a reliable measure for fetal exposure. The use of deciduous teeth provides a unique opportunity to directly measure fetal exposure. Furthermore, the ability to *retrospectively* reconstruct prenatal and early childhood exposure would permit temporal exposure information to be ascertained in case-control studies. This, in our opinion, is likely to improve efficiency in the study of diseases that occur at lower frequency and manifest during childhood and adolescence (autism spectrum disorders, for example).

Whole teeth and fragments of dentine have been successfully used in many studies to estimate cumulative Pb exposure [9], [10], [21]. Needleman and colleagues [9] observed that children who lived in environments with greater risk of Pb exposure had higher dentine Pb levels than children who lived in areas with lower Pb exposure. Further validation was undertaken by Rabinowitz and colleagues who showed that dentine Pb measurements and blood Pb concentrations were significantly correlated [10]. Subsequently, dentine and whole tooth Pb levels were used in studies of childhood neurodevelopment and this Pb biomarker was found to be associated with several developmental parameters including IQ [8], [21]. Gulson and colleagues advanced these methods by means of stable Pb isotope analysis that allowed identification of source of Pb exposure [7]. With the advent of micro-spatial analytical methods, including laser ablation and proton- and synchrotron-induced X-ray emission methods, it is now possible to take advantage of the incremental structure of teeth to reconstruct the timing of Pb exposure. However, before micro-spatial dentine Pb analysis can be applied to the study of health outcomes, it is important that this method is validated against other exposure biomarkers.

Much work has been undertaken on the kinetics of Pb in blood which is why it has been the marker of choice to explore the adverse health effects of Pb exposure in most epidemiologic studies. The application of K-XRF analytical methods to the in vivo measurement of Pb levels in bone provided a way to study cumulative Pb exposure, which cannot be obtained from single blood Pb measurements due to the short half-life of Pb in blood [17]. Therefore, to validate dentine Pb as a biomarker of the timing and intensity of Pb exposure, we compared dentine Pb with these established biomarkers.

When using the dentine-Pb biomarker to uncover prenatal exposure, we found that Pb levels in sampling points in dentine at the neonatal line were positively associated with cord blood Pb concentrations. Our laser sampling points are 35 µm in diameter which represent exposure over approximately 12–15 days around birth. Because cord blood Pb represents levels in fetal circulation in the latter part of the third trimester, a strong positive correlation between these two matrices supports our hypothesis that dentine Pb is a suitable marker of prenatal exposure. However, to confirm that sampling points in dentine capture the timing of prenatal Pb exposure we undertook additional analyses. We compared Pb levels in dentine sampling points at the neonatal line with Pb concentrations in blood collected at different ages in childhood. It was our hypothesis that if dentine indeed captures the *timing* of Pb exposure, then measurements in dentine formed at birth would show the strongest correlation with cord blood Pb and a progressively weaker association with circulating Pb levels at older ages. Our observations (Fig. 2b) confirmed this hypothesis.

Beyond the analysis of dentine formed at the time of birth, we wanted also to check the utility of our biomarker in predicting cumulative prenatal exposure. Lead stored in maternal bones is mobilized to maternal circulation during pregnancy and lactation and serves as an important source of fetal Pb exposure [18]. Bone Pb measurements were undertaken at 1 month postpartum in our study and have been applied effectively to study prenatal Pb exposure and childhood health in this cohort [19]. When we compared Pb levels in prenatal dentine (as area under curve of all prenatal sampling points), we found a significant positive association with both patella and tibia Pb measurements. The stronger association with patella Pb is not surprising because Pb deposits in trabecular bone (predominant component of patella) are comparatively more mobile and exchange more readily with plasma than Pb stored in cortical bone (predominant component of tibia) [17], [18]. Similarly, we found that Pb levels in dentine formed during the second or third trimesters were significantly associated with Pb concentrations in maternal blood collected concurrently.

An important feature of secondary dentine is that it continues to accrue Pb and other elements as long as the tooth remains vital. It is, therefore, a potential target to measure integrated life-time exposure up to the time the tooth is shed (or no longer depositing dentine). We examined secondary dentine in our tooth sections, avoiding tertiary dentine deposits which may corrupt our analysis, and measured average Pb concentrations over seven sampling points. We compared this measure to the CBLI which integrates blood Pb concentrations over different ages and is an established measure of cumulative Pb exposure [11], [12]. In this analysis we used data from children who had blood Pb measurements from the ages of 1.5 to 6 years, making this one of the most detailed analyses of secondary dentine Pb with serial blood Pb measurements undertaken to date. We observed a significant positive association between the two measures suggesting that average Pb concentrations in secondary dentine do provide a measure of long-term cumulative exposure.

The main limitation of our study is that we did not have a complete set of biomarkers on all 85 participants due to incomplete follow-up and sample collection. However, our sample size was sufficient to test our hypotheses and uncover statistically significant associations between respective biomarkers. Secondly, we have used data from the first 85 participants who donated teeth. To examine differences in exposure profile of our participants from the wider ELEMENT cohort, we compared the Pb levels in maternal blood and bone to those reported from earlier work on the ELEMENT participants. We only observed minor differences (compare data in Table 2 with data in Tellez-Rojo et al. [14] and Hu et al. [22]). Another limitation of our study is that we do not have multiple teeth from each participant. Consequently, we are unable to explore if tooth type influences dentine Pb concentrations. Ideally, within each individual, we should compare Pb levels in multiple teeth in dentine formed at the same time (at birth, for example), thereby parsing out the influence of tooth type from differences in exposure.

Metal concentrations in teeth have served as exposure biomarker in many studies. With modern techniques that allow micro-spatial measurement of multiple metals in teeth, we now have the opportunity to reconstruct past exposure history not only in terms of cumulative exposure but also for the timing of exposure. This will allow us to examine critical windows of susceptibility even when those windows occur prenatally, which would be a major advance in environmental epidemiology considering that maternal exposure biomarkers do not always represent fetal exposure adequately. A barrier in the application of this biomarker to the study of health outcomes has been the lack of appropriate validation of this methodology. In the present study, we have shown that measurements of Pb in discrete regions of dentine can be reliably used to reconstruct Pb exposure history over the prenatal and early childhood periods and secondary dentine holds the potential to estimate long-term exposure up to the age of tooth shedding.

## Supporting Information

Table S1 Brief description of anatomical terms used in this manuscript.
(DOCX)
