# Supplementary material for: Determining Prenatal, Early Childhood and Cumulative Long-Term Lead Exposure Using Micro-Spatial Deciduous Dentine Levels
Source: PLoS One. 2014 May 19;9(5):e97805. doi: 10.1371/journal.pone.0097805 (PMC4026445; doi:10.1371/journal.pone.0097805)
Supplement: Table S1 — Brief description of anatomical terms used in this manuscript. (DOCX) [file pone.0097805.s001.docx]

**Supporting Information S1**

**Table S1.** Brief description of anatomical terms used in this manuscript [1, 2].

| **Structure** | **Description** |
| --- | --- |
| Enamel dentine junction | Boundary between enamel and dentine (also see Figure 1) |
| Prenatal dentine | Dentine formed before birth (identified using neonatal line in this study) |
| Postnatal dentine | Dentine formed after birth |
| Primary dentine | Dentine that forms the bulk of the tooth and outlines the secondary dentine and pulp chamber |
| Secondary dentine | Dentine formed after root formation has been completed. It lies between primary dentine and pulp chamber |
| Tertiary dentine | Dentine formed in response to external stimuli (e.g. caries, attrition). It is formed adjacent the pulp in areas affected by the stimulus |
| Coronal dentine | Dentine located in the crown of the tooth |
| Radicular dentine | Dentine located in the root of the tooth |
| Cervical margin | Junction between crown and root of teeth |
| Pulp chamber | Central compartment of tooth which is surrounded by dentine and where the neurovascular tissue is located |

1. Berkovitz BKB, Holland GR, Moxham BJ (2009) Oral Anatomy, Histology and Embryology: Elsevier.
2. Ten Cate A.R. (1998). Oral Histology: development, structure and function. 5^th^ ed. Mosby.
